# Flow-through Gas Phase Photocatalysis Using TiO_2_ Nanotubes on Wirelessly Anodized 3D-Printed TiNb Meshes

**DOI:** 10.1021/acs.nanolett.3c01149

**Published:** 2023-07-12

**Authors:** Hanna Sopha, Adelia Kashimbetova, Michal Baudys, Pavan Kumar Chennam, Marcela Sepúlveda, Jakub Rusek, Eva Kolibalova, Ladislav Celko, Edgar B. Montufar, Josef Krysa, Jan M. Macak

**Affiliations:** † Center of Materials and Nanotechnologies, Faculty of Chemical Technology, University of Pardubice, Nam. Cs. Legii 565, 53002 Pardubice, Czech Republic; ‡ Central European Institute of Technology, Brno University of Technology, Purkynova 123, 612 00 Brno, Czech Republic; § Department of Inorganic Technology, University of Chemistry and Technology Prague, Technicka 5, 166 28 Prague, Czech Republic

**Keywords:** Nb-doped TiO_2_ nanotube layers, bipolar electrochemistry, TiNb mesh, 3D printing, direct ink writing, photocatalysis

## Abstract

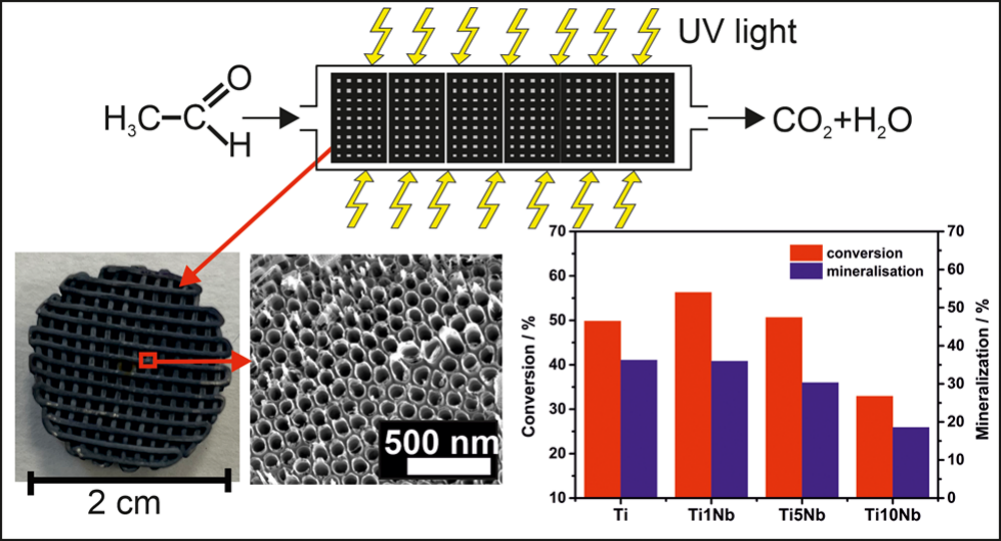

In this work, for the first time 3D Ti-Nb meshes of different composition, i.e., Ti, Ti-1Nb, Ti-5Nb, and Ti-10 Nb, were produced by direct ink writing. This additive manufacturing method allows tuning of the mesh composition by simple blending of pure Ti and Nb powders. The 3D meshes are extremely robust with a high compressive strength, giving potential use in photocatalytic flow-through systems. After successful wireless anodization of the 3D meshes toward Nb-doped TiO_2_ nanotube (TNT) layers using bipolar electrochemistry, they were employed for the first time for photocatalytic degradation of acetaldehyde in a flow-through reactor built based on ISO standards. Nb-doped TNT layers with low concentrations of Nb show superior photocatalytic performance compared with nondoped TNT layers due to the lower amount of recombination surface centers. High concentrations of Nb lead to an increased number of recombination centers within the TNT layers and reduce the photocatalytic degradation rates.

Semiconductors are of high interest in the field of photocatalysis, e.g., for the photocatalytic degradation of pollutants. Especially TiO_2_ is frequently used for such applications, as it has proven to be an excellent and stable photocatalyst with low production costs.^1^ However, TiO_2_ has some drawbacks, such as a high bandgap of 3.2 eV enabling just the absorption of UV light and a high amount of electron–hole recombination centers. Therefore, TiO_2_ is often doped with transition metals, such as W, Co, Fe, Mo, or Nb, to increase its efficiency as photocatalyst.^2−5^ Nb doping was shown in several publications to enhance the photocatalytic activity for the degradation of pollutants.^2−8^ Nb^5+^ can substitute Ti^4+^ in the TiO_2_ lattice, adding an additional valence electron, and therefore, it acts as a donor atom. Charge compensation can be achieved either by cation vacancies or by stoichiometric reduction of Ti^4+^ to Ti^3+^.^2,9^

Utilization of a nanostructured photocatalyst significantly increases the surface area of the catalyst, which results in a higher efficiency. Among many different TiO_2_ nanostructures, as for instance nanoparticles, nanorods, nanofibers and nanotubes, TiO_2_ nanotube (TNT) layers produced via anodization have attracted enormous attention within the past 20 years.^10,11^ The advantages of such TNT layers over TNTs produced in powder form by other methods (e.g., hydrothermally) are their vertical alignment resulting in a high degree of order, their strong interconnection, and their connection to the underlying Ti substrate, enabling their use without any further immobilization. Additionally, by anodizing Ti alloys, metal doped TNT layers can easily be fabricated.^12−16^ Since the first reports on the anodization of TiNb alloys,^13,17^ such Nb-doped TNT layers have been shown to be very efficient in many different applications, such as dye-sensitized solar cells (DSSC),^18^ photocatalysis,^19^ biomedical applications,^20^ or the photocatalytic CO_2_ conversion toward acetaldehyde.^21^

Within the last years, more complicated Ti substrates, such as meshes,^22−25^ wires,^26−28^ or spheres,^29^ have been employed for TNT layer fabrication, maximizing the anodized surface area for catalytic applications. Among others, such more complicated 3D Ti substrates can be produced using additive manufacturing. However, though additive manufacturing offers the fabrication a plethora of different shapes, rather few studies have shown their modification with TNT layers.^25,30−37^ These have been mainly pure Ti and biomedical Ti6Al4V alloy with the aim of their application as implants.^30−36^ However, also TiNb-based alloys have been shown to be producible using additive manufacturing, by using either selective laser melting (SLM)/laser-based powder bed fusion (LB-PBF),^38−44^ or laser engineered net shaping (LENS)/laser directed energy deposition.^45,46^ Direct ink writing (DIW) has so far not been used for the production of TiNb alloys, although DIW has the advantage of simple powder blending, microstructural control through the sintering regimen, and using just the amount of metal powder needed for printing, thus reducing the environmental footprint and production cost.^47^

The anodization of such complicated 3D Ti based structure toward their modification with TNT layers is rather challenging as the high surface area to be anodized increases the chance of dielectric breakdown.^48,49^ Furthermore, for the complete anodization, the 3D structure must be fully inserted into the electrolyte, while a connection to the potentiostat must be established. This is almost impossible in the case of spheres or other solid structures. In the case of meshes or hollow structures, a connection consisting of a thin Ti wire (other materials would contaminate the electrolyte by the release of other metal ions during anodization) would theoretically be possible. However, such a thin connection would also be very prone to dielectric breakdown at the electrolyte/air interface and is therefore unsuitable. Recently, we demonstrated the possibility of wireless anodization of Ti spheres and 3D printed Ti meshes to overcome these challenges.^25,29^ Under the regime of bipolar electrochemistry with alternating potential, TNT layers were produced on the whole surface of the 3D structures without a connection to the potentiostat due to the polarization of the Ti substrate in a high electrical field between two feeder electrodes.

In the present work, 3D Ti–Nb meshes were prepared for the first time via DIW using Ti–Nb powder mixtures with nominal compositions of Ti-1Nb, Ti-5Nb, and Ti-10Nb. Subsequently, these meshes were anodized using bipolar electrochemistry to grow TNT layers on their surface and finally used for the photocatalytic degradation of acetaldehyde in a flow-through gas phase reactor. The meshes had a diameter of 20 mm and a height of 8 mm and consisted of an orthogonal Cartesian grid pattern with a filament distance (i.e., pore size in the printing plane) of 857 ± 26 μm resulting in a porosity of 68 ± 1%.^25^ No statistically significant differences in pore size and porosity were observed between the mesh compositions; therefore, the former values correspond to averages and standard deviations for all meshes produced. The effective surface area of each mesh was calculated to ∼61 cm^2^ (equivalent to 2.4 mm^2^/mm^3^), giving a very high surface to volume ratio.^25^ EDX analysis revealed Nb contents within the as-prepared 3D meshes between 60 and 80% of the nominal composition (Table S1). Figure S1 shows the SEM images of the as-prepared 3D meshes, revealing that Ti and Nb powders underwent interdiffusion during sintering, densifying the filaments while forming the binary TiNb alloy. The produced meshes of all substrates possess rough surfaces and large grains. There are globular particles discernible on the surface, together with stratifications, that stem from the surface mass transport during sintering. The high roughness of the 3D Ti meshes was already shown in our previous work.^25^ Furthermore, the TiNb meshes had a biphasic lamellar microstructure of beta- (β-) and alpha- (α-) Ti, with the number and thickness of β-Ti lamellas (solid solution of Nb in Ti) increasing with the increment of Nb in the alloy, while, in contrast, the reference pure Ti mesh had a monophasic microstructure of equiaxed α-Ti grains (Figure S1).

As this was the first time that TiNb alloys were prepared via DIW, the stress–strain response of the printed alloys was investigated. As one can see in Figure S2, the incorporation of Nb into the Ti meshes roughly doubled the compressive strength of the 3D meshes for all three Nb contents. The effective elastic modulus, on the other hand, increased monotonically with the Nb content, meaning that the 3D meshes become stiffer with the addition of Nb. The 3D Ti and Ti-10Nb meshes showed an abrupt drop in stress and a quasi-brittle fracture soon after the elastic regime. In contrast, the 3D Ti-1Nb and Ti-5Nb meshes showed a serrated and slow decline in stress after the maximum strength due to mesh densification allowed by the ductility of the alloys. Therefore, the incorporation of Nb increases the ductility of Ti, but excessive formation of β-Ti lamellas reduces the ductility without the reduction of the compressive strength. Generally, all prepared 3D meshes were mechanically robust and allowed for the flow of fluids without structural damage, showing their potential application in self-supported flow-through systems.

Before further use, the 3D meshes were characterized by using X-ray diffraction (XRD). Figure 1A depicts the XRD patterns for all 3D meshes. 3D Ti and Ti-1Nb meshes show purely α-Ti peaks (PDF 00-005-0682), while β-Ti peaks (PDF 03-065-5970) were found for 3D Ti-5Nb and Ti-10Nb meshes. Rietveld refinement was used to calculate the phase fractions of β-Ti to 9.0 and 22.9% for the Ti-5Nb and Ti-10Nb meshes. The Nb content was too low to be detected using XRD. Moreover, the measurements show that the 3D meshes were not visibly contaminated with other metals.

**Figure 1:**
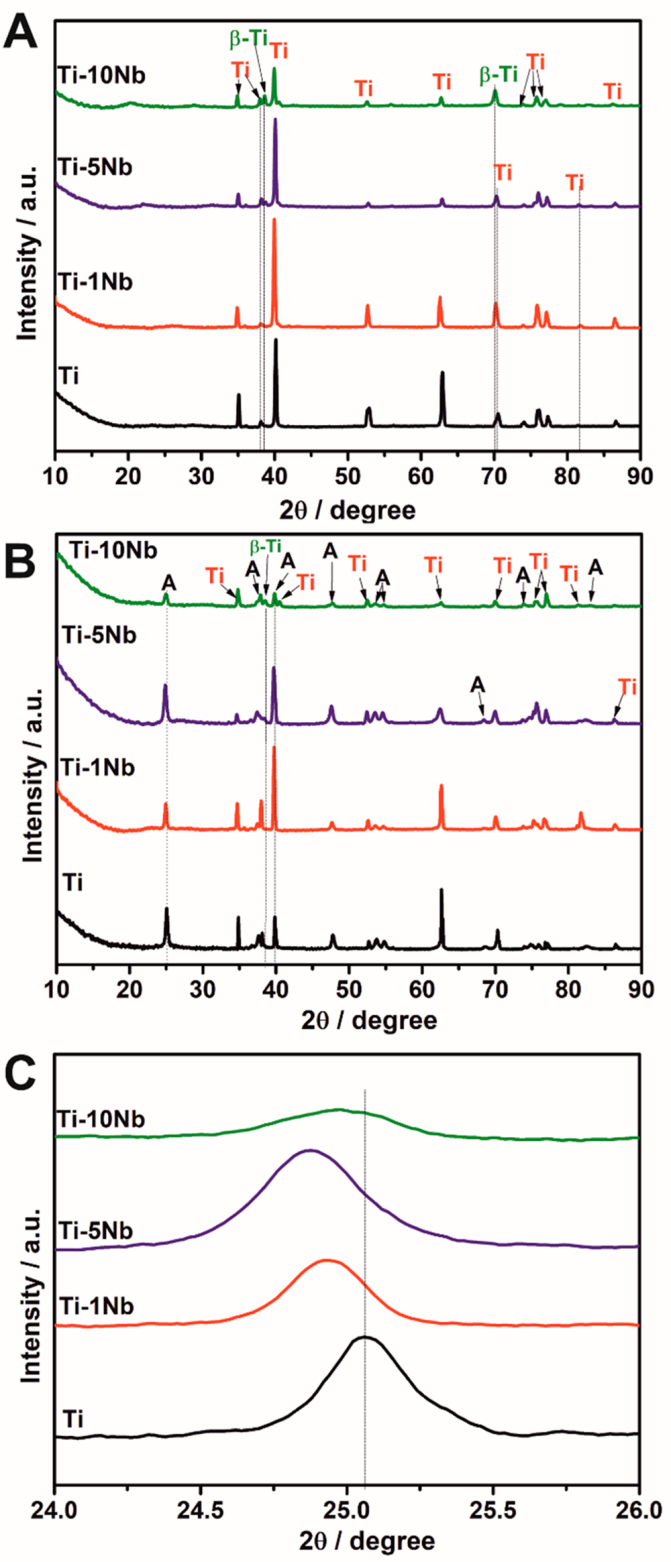
XRD patterns of A) as-prepared and B) TNT layer modified 3D Ti and TiNb alloy meshes. C) The shift of the anatase peak for the anodized 3D meshes. Ti = α-titanium, β-Ti = β-titanium, A = TiO_2_ anatase.

The 3D meshes were further wirelessly anodized toward TNT layers in an ethylene glycol-based electrolyte containing 170 mM NH_4_F and 1.5 vol % H_2_O using a bipolar electrochemical setup.^25^ Afterward, the 3D meshes were annealed at 400 °C for 1 h to convert produced amorphous TNT layers into the TiO_2_ anatase phase. XRD patterns after anodization and annealing are shown in Figure 1B and reveal additionally α-Ti and β-Ti peaks, stemming from the underlying 3D meshes, TiO_2_ anatase peaks (PDF 01-076-8999) with the main peak at 2θ = 24.95° corresponding to the (101) orientation.

Nb_2_O_5_ was not observed within the anodized 3D meshes. This is not anyhow surprising, as it was shown in the literature that even XRD patterns of anodized Ti-45Nb alloys annealed at 450 °C did not show any Nb_2_O_5_ peaks, although the Nb content was significantly higher as herein.^13^ Just after annealing at 650 °C, a very small Nb_2_O_5_ peak was detected in the mentioned study.^13^

However, in Figure 1B, a slight shift of the anatase (101) peak at 2θ ∼ 25° to lower 2θ values can be observed for the 3D TiNb meshes compared to that for the pure 3D Ti meshes. For clarity, Figure 1C shows a magnification of the peak. The reason for this peak shift is the similar atomic radius of Ti^4+^ and Nb^5+^ (i.e., 0.605 Å vs 0.64 Å), which results in an easy replacement of Ti^4+^ with Nb^5+^ species in the lattice. This increases the lattice spacing and decreases the diffraction peak position. Thus, the diffraction peak shift suggests a doping of Nb^5+^ into the anatase lattice.^19^

The surface chemical composition of the anodized 3D meshes was evaluated by using X-ray photoelectron spectroscopy (XPS). The survey spectra for all four employed 3D meshes are shown in Figure 2A. In all TNT layers, the presence of Ti, O, and C was detected; however, Nb was not found, likely because it has leached out from the uppermost surface of the nanotube layer (due to dissolution into the electrolyte). Figure 2B and 2C show the Ti 2p and O 1s high-resolution (HR) spectra for all 3D meshes, respectively. The Ti 2p region is well-defined for the TNT layers on all 3D meshes and presented characteristic spin–orbit components observed at of 458.6 eV (Ti 2p_3/2_) and 464.3 eV (Ti 2p_1/2_), resulting in the components energy separation (Δ) of 5.7 eV. Therefore, the Ti 2p spectra features indicate the presence of the Ti^4+^–O bond in TiO_2_.^50,51^ At the same time, the O 1s peak of the analyzed TNT layers shows two contributions centered at 529.9 and 531.3 eV. These peaks can be attributed to the bonds of titanium oxygen (Ti–O)^50,51^ and hydrogen–oxygen (Ti–OH),^51−53^ respectively. No bonds attributed to other compounds were detected. The atomic concentrations of Ti, O, and C were calculated from the HR spectra and are given in Table S2. The stoichiometry was calculated from the concentrations of Ti and O for all four 3D meshes, resulting in O:Ti ratios of 2.17, 2.19, 2.2, and 2.27 for the 3D Ti, Ti-1Nb, Ti-5Nb, and Ti-10Nb meshes, respectively. This suggests the possibility of an increased Nb concentration within the TNT layers formed on Ti-10Nb compared to that on Ti-1Nb and Ti-5Nb.

**Figure 2:**
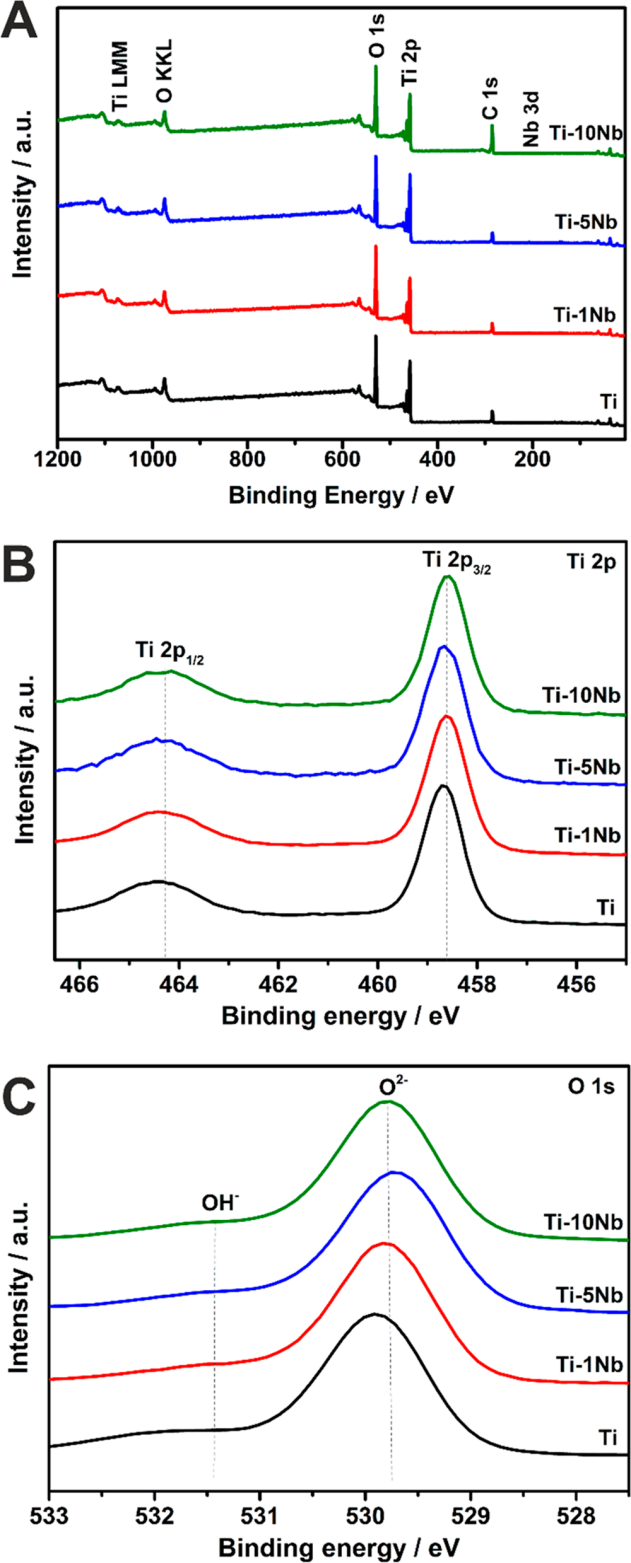
XPS spectra of the Ti and TiNb alloy 3D meshes: A) survey spectra, B) high-resolution Ti 2p spectra, and C) high-resolution O 1s spectra.

To get more insights into the Nb content, extensive TEM/HRTEM/STEM/EDX analyses were carried out on the nanotubes grown on the 3D Ti-1Nb and Ti-10Nb meshes, as shown in Figure S4. As one can see, Nb was found in nanotubes grown on both meshes, with a significantly lower amount of Nb in the nanotubes grown on the Ti-1Nb mesh. However, it must be noted that Nb was just found in nanotube fragments with thick walls, stemming from the bottom of the original nanotubes, but not in nanotubes with thin walls from the tops. This confirms the assumption that the Nb is leached out on the nanotube tops due to heavy etching.

SEM top-view images shown in Figure 3 demonstrate that TNT layers were indeed produced on all 3D meshes. The SEM top view images were taken on the top of the outer filaments of the 3D meshes where the potential is the highest. An uneven potential distribution along the 3D meshes, due to the convenient use of bipolar electrochemistry for anodization, results in TNTs with a gradient in diameter and thickness from the outer parts toward the middle of the meshes.^25,29,54,55^ The diameters of the TNTs on the outermost filaments were measured to be 84.7 ± 9.1, 57.5 ± 7.0, 89.8 ± 20.5, and 78.9 ± 13.4 nm for the 3D Ti, Ti-1Nb, Ti-5Nb, and Ti-10Nb meshes, respectively. It must be noted here that on surfaces of all 3D meshes large amounts of nanograss were found,^56^ as shown in Figure S3 for an anodized 3D Ti-5Nb mesh. This nanograss stems from an etching of the TNT surface during the anodization process in strong electrolytes (i.e., high F-content), at high potentials (resulting in high current densities), and during long anodization times, leading to a thinning and partial disintegration of the TNT walls. Major parts of this nanograss can be removed by prolonged sonication of the 3D meshes in isopropanol after anodization; however, some remnants stay on the TNT surface.

Thickness measurements of the TNT layers were carried out on SEM cross-sectional images prepared by carefully scratching TNT layers from the 3D meshes to carbon tape located on SEM stubs. The thickness of the TNT layers varied significantly on all different 3D meshes, ranging on each individual mesh from ∼1.5 to ∼7 μm. The reason for this is 2-fold: (i) due to the strong etching of the TNT layer surface and the formation of nanograss, some parts of the TNT layers were significantly shortened compared to others, and (ii) due to the use of bipolar electrochemistry, every curved filament underwent locally different potentials on different parts depending on the position toward the feeder electrodes. However, as on 3D Ti and TiNb meshes of all compositions, TNT layer thicknesses in the same range were found, it is expected that the TNT layers grow equally and in the same thickness on 3D meshes with all studied compositions (Figure 3).

**Figure 3:**
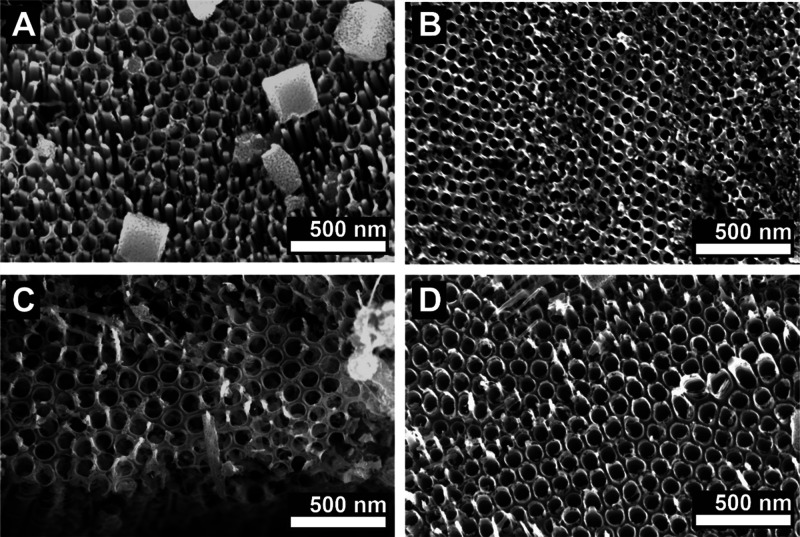
SEM top-view images of TNT layers prepared on 3D A) Ti, B) Ti-1Nb, C) Ti-5Nb, and D) Ti-10Nb meshes.

The anodized and annealed 3D meshes were further used for gas-phase photocatalysis using acetaldehyde as a model pollutant, proving the 3D meshes as self-supported and high-performance photocatalytic substrates. A scheme of the reactor used, built according to ISO standards (ISO 22197-2), is shown in Figure S5. It consisted of a quartz glass tube with an inner diameter of 22 mm, in which six 3D meshes were stacked, surrounded by 12 UV lamps (8 W each, λ_max_ = 365 nm). Acetaldehyde was mixed with synthetic air with 50% humidity to a concentration of 5 ppm. After a stable concentration was reached, the gas flow was directed through the reactor in the dark. The UV light was turned on after 40 min, when an adsorption equilibrium was reached, to induce the photocatalytic degradation of acetaldehyde.

As a first step, the optimal flow rate of acetaldehyde through the reactor was determined using anodized 3D Ti meshes as photocatalyst. The dependency of the acetaldehyde conversion and mineralization (full degradation of acetaldehyde to CO_2_, following eq 1(57)) are depicted in Figure 4 and Figure S6.
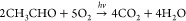
1

**Figure 4:**
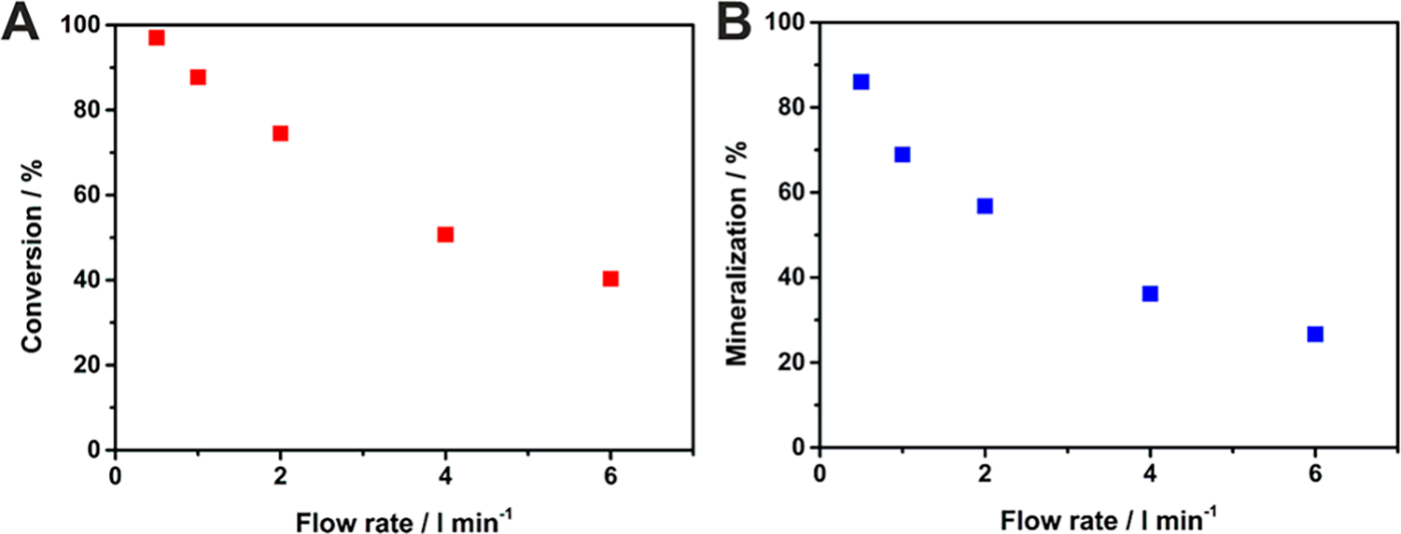
Dependency of acetaldehyde A) conversion and B) mineralization on the flow rate of acetaldehyde through the reactor, using 3D Ti meshes as photocatalyst.

As one can see from Figure 4, the highest conversion and mineralization values of ∼97 and ∼86%, respectively, were obtained for the slowest flow rate of 0.5 l/min due to a long residence time of acetaldehyde within the reactor, allowing an intensive contact with the 3D Ti mesh photocatalyst, while for the highest tested flow rate of 6 l/min conversion and mineralization dropped to ∼40 and ∼27%, respectively. For further measurements, a flow rate of 4 l/min was chosen with a conversion of ∼50% to observe differences in conversion and mineralization for the 3D meshes of different composition.

Figure 5 shows conversion and mineralization of acetaldehyde for all anodized 3D meshes (including pure 3D Ti meshes). The highest conversion and mineralization of acetaldehyde was received for 3D Ti-1Nb alloy meshes with a conversion of ∼56% and a mineralization of ∼36%. The 3D Ti and Ti-5Nb meshes both showed a conversion of ∼50% and a mineralization of 36% and 30%, respectively, while the 3D Ti-10Nb meshes showed the lowest conversion and mineralization with ∼33% and 19%, respectively. The increase of the photocatalytic activity of the anodized 3D Ti-1Nb meshes compared to the 3D Ti meshes can be explained with the Nb doping of the TNTs and an introduction of defects into the TNT crystalline lattice by a reduction of Ti^4+^ to Ti^3+^.^2,9^ However, if the Nb content within the TNTs increased, the surface states induced by Nb might also act as recombination centers for electron–hole pairs.^58,59^ Therefore, 3D Ti-5Nb and Ti-10Nb meshes are less favorable for the photocatalytic degradation of acetaldehyde.

**Figure 5:**
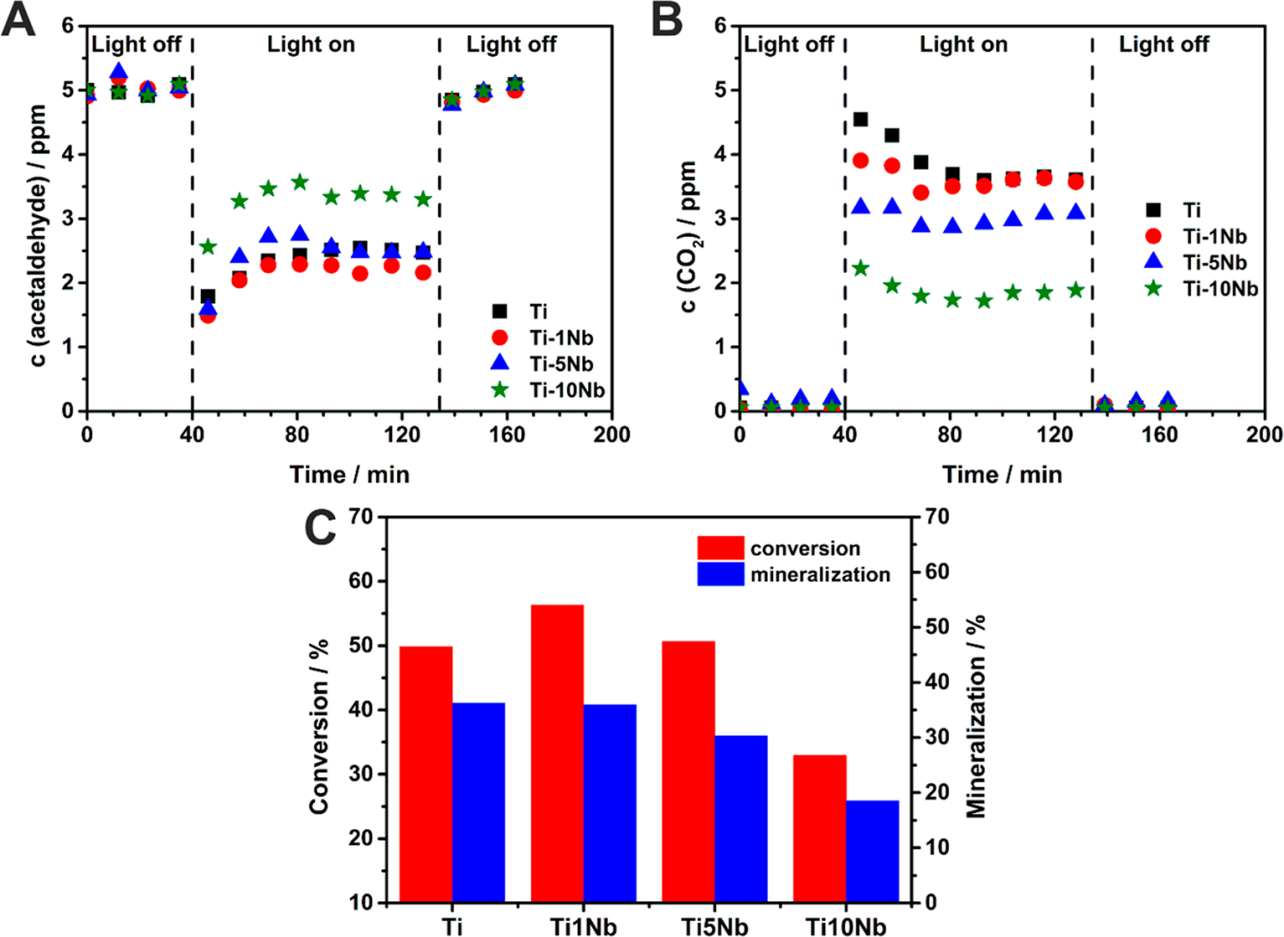
Photocatalytic changes in A) acetaldehyde concentration, B) CO_2_ production on 3D Ti and TiNb alloy meshes. C) The conversion and mineralization in percent. Flow rate 4 l/min.

For comparison, nonanodized, annealed 3D meshes, i.e., 3D meshes covered with a thin thermal TiO_2_ layer, but without TNT layers, were also investigated as photocatalysts for the photocatalytic degradation of acetaldehyde. The results are shown in Figure S7. In fact, no conversion of the acetaldehyde was observed. This can be explained with the significantly smaller surface area of the 3D meshes without TNT layers compared to their TNT layer modified counterparts, showing that the large surface area of the TNT layers is of paramount importance for the photocatalytic degradation of pollutants in gas phase flow-through reactors.

In summary, the use of DIW to produce mechanically robust 3D Ti and TiNb alloy meshes suitable for self-supporting flow-through catalytic systems was shown for the first time. The incorporation of Nb doubled the mechanical strength of the 3D meshes, while their wireless anodization using bipolar electrochemistry created a high surface area and highly active nanotubular photocatalyst. The possible use of such TNT layer modified 3D meshes in a flow-through photocatalytic reactor was proven for the degradation of acetaldehyde, showing the great potential of the 3D meshes. Anodized 3D Ti-1Nb meshes showed the highest photocatalytic activity due to the reduction of the bandgap through the introduction of defects into the TNT crystalline lattice at a minimum formation of electron–hole recombination centers. The results presented herein positively show the possibility of employing additive manufacturing for building up robust 3D networks that can be used in flow-through photocatalytic systems after nanostructuring their surfaces.

## Supporting Information Available

Methods, EDX measurements, atomic concentration deduced by XPS, SEM images of nonanodized 3D meshes, mechanical performance of the 3D meshes, additional SEM images of the anodized 3D Ti-5Nb mesh, scheme of the gas phase photocatalytic reactor, photocatalytic degradation and mineralization of acetaldehyde using 3D Ti meshes as photocatalyst for different flow rates of acetaldehyde, photocatalytic degradation and mineralization of acetaldehyde on annealed 3D Ti and TiNb alloy meshes without TNT layers (PDF)
