# Supplementary material for: Flow-through Gas Phase Photocatalysis Using TiO2 Nanotubes on Wirelessly Anodized 3D-Printed TiNb Meshes
Source: Nano Lett. 2023 Jul 12;23(14):6406–13. doi: 10.1021/acs.nanolett.3c01149 (PMC10375580; doi:10.1021/acs.nanolett.3c01149)
Supplement: Supplementary file 1 — nl3c01149_si_001.pdf [file nl3c01149_si_001.pdf]

# Supporting Information

# Flow-through gas phase photocatalysis using $TiO_2$ nanotubes on wirelessly anodized 3D-printed TiNb meshes

*Hanna Sopha[†,‡], Adelia Kashimbetova[‡], Michal Baudys[§], Pavan Kumar Chennam[‡], Marcela Sepúlveda[†], Jakub Rusek[§], Eva Kolibalova[‡], Ladislav Celko[‡], Edgar B. Montufar[‡], Josef Krysa[§], Jan M. Macak[†,‡,]***

[†]Center of Materials and Nanotechnologies, Faculty of Chemical Technology, University of Pardubice, Nam. Cs. Legii 565, 53002 Pardubice, Czech Republic

[‡]Central European Institute of Technology, Brno University of Technology, Purkynova 123, 612 00 Brno, Czech Republic

[§]Department of Inorganic Technology, University of Chemistry and Technology Prague, Technicka 5, 166 28 Prague, Czech Republic

*Corresponding author: jan.macak@upce.cz

**Methods**

3D meshes with four different compositions prepared via direct ink writing[1,2] were employed in this study, namely Ti, Ti-1Nb, Ti-5Nb, and Ti-10Nb meshes. To prepare them, 0, 1, 5, and 10 wt.% pure niobium powder (<45 µm, 99.8 % (trace metals basis); Sigma-Aldrich, USA) were added to commercially pure spherical Ti powder (20−63 µm, ASTM Grade 1; TLSTechnik, Germany) and homogenized for 3 h using a laboratory roll mixer at a speed of 50 rpm. Afterwards, the powders were mixed with gelatine as a water-soluble organic binder. Immediately after mixing, the inks were introduced into the cartridge of the robotic deposition device and the Ti and Ti-Nb meshes were printed in air at a speed of 20 mm/s following an orthogonal deposition pattern of linear filaments in consecutive layers with an infill density of 31 %. After printing, the meshes were dried at room temperature for 12 h and then heated in a muffle furnace in air to 350 °C for 12 h to remove the organic binder, and finally sintered in argon atmosphere at 1400 °C for 10 h. The sintered meshes had a diameter of 20 mm, a height of 8 mm, filaments of 543 ± 17 µm in diameter, separation between filaments of 857 ± 26 µm, porosity of 68 ± 1 % and a surface area of 61 $cm^2$ (equivalent to 2.4 $mm^2/mm^3$) each.

Compression tests (n = 4 per series) were performed using a servohydraulic testing system (Instron 8874; USA) with a 10 kN load cell at a crosshead speed of 1 mm/min, applying the load perpendicularly to the printing plane. Cubic meshes fabricated as described in the previous paragraph were tested. The lateral faces of the meshes were cut with a diamond disc to avoid edge effects and the top and bottom surfaces were carefully polished to ensure flat parallel surfaces, achieving a final side length of ~10 mm. The compressive strength (peak value) and effective elastic modulus (slope of the linear loading segment) were obtained from recorded stress–strain curves corrected for machine load chain compliance. The cross-section area for the stress calculation was the area of the cube base calculated for each mesh from the external dimensions.

Before further use, the meshes were sonicated in isopropanol and acetone for 1 min each, rinsed with isopropanol, and dried in air.

The nominal composition of the 3D Ti-Nb meshes was determined before further treatment using energy dispersive spectroscopy (EDX), carried out using a scanning electron microscope (SEM, LYRA3, Tescan, equipped with EDX analyzer AZtec X-Max 20, Oxford Instruments) using an accelerating voltage of 20 kV. On each 3D Ti-Nb mesh five measurements were carried out on the as-prepared surfaces and the results are given as average ± standard deviation (SD). The results are given in **Table S1**.

The 3D meshes were anodized at 120 V in an ethylene glycol-based electrolyte containing 176 mM $NH_4F$ and 1.5 wt.% $H_2O$ using a bipolar set-up with alternating potential (frequency: 5.55 × $10^{-4}$ Hz), as described in detail in our previous work.[2] Afterwards, all 3D meshes were annealed in a muffle oven at 400 °C for 1 h using a heating rate of 2.1 °C/min.
The morphology of the as-prepared, as well as anodized and annealed 3D meshes was characterized using a field emission scanning electron microscope (FE-SEM, FEI Verios 460 L). Metallographic cross-sections of the as-prepared meshes were obtained following standard methods (grinding and polishing), with final polishing using colloidal silica suspension. Cross-section images to investigate the TNT layer thickness were obtained by carefully scratching the TNT layers. Statistics on nanotube diameter and TNT layer thickness were carried out using proprietary Nanomeasure software. The results are given as average ± standard deviation (SD). Transmission electron microscopy (TEM) analysis was carried out using a Titan Themis 60–

300 (Thermo Fisher Scientific) instrument. The instrument operated at 300 kV and was equipped with a high angle annular dark field detector for scanning transmission electron microscopy (HAADF-STEM) and SUPER-X energy dispersive X-ray (EDX) spectrometer with 4×30mm$^2$ windowless silicon drift detectors. To receive fragments of single TiO2 nanotubes (transparent for electrons), the TNT layers were removed from the underlying meshes by very strong ultrasonication. The nanotube fragments were analyzed on a Cu grid.

X-Ray Diffraction (XRD, SmartLab 3 kW diffractometer Smartlab from Rigaku (Japan)) measurements were carried out with Bragg-Brentano geometry, Cu-α radiation (λ = 0.154 nm), and a Dtex-Ultra 1D detector. The Cu lamp radiation was powered by a current of 30 mA and a voltage of 40 kV. A step size of 0.02° and a scanning speed of 4°/min was used, and the XRD patterns were recorded in the range from 10° to 90°. PDXL2 software was used for evaluation of the patterns.

The surface chemical composition of the TNT layer modified meshes was performed using X-ray photoelectron spectroscopy (XPS; Kratos Axis Supra) with a monochromated Al Kα X-ray source (hυ = 1486.69 eV) at an emission current of 15 mA with hybrid lens mode. Fitting and peak deconvolution were carried out using CasaXPS software (Version 2.3.17, Casa Software Ltd., Teignmouth, UK). High-resolution (HR) spectra were used to determine the elemental composition and chemical states. The quantification of HR peaks was conducted applying Shirley background deduction and the sensitivity factors were provided by the manufacturer. Prior to data analysis, the binding energy scale was corrected by means of C 1s spectrum at 284.8 eV (adventitious carbon). As an initial perception, the presence of Ti 2p, O 1s, and C 1s peaks is identical in the survey spectra of all samples with TNT layers. The presence of C 1s is a measurement artefact and is associated with adventitious carbon.[3]

The photocatalytic degradation of acetaldehyde was carried out in an apparatus based on ISO standard 22197-2 (removal of acetaldehyde). The set-up is shown in **Figure S4**. The reactor consisted of a quartz tube with an inner diameter of 22 mm, equipped with two Teflon flange enabling to connect it to 6 mm Teflon tubes. Six 3D meshes were placed in the middle of the quartz tube as photocatalyst. Twelve fluorescent tubes with a power output of 8 W each (emission maximum 365 nm (NBB)) were placed around the quartz tube and used as UV light source. The concentration of acetaldehyde was analysed using a gas chromatograph (GC 7890B, Agilent) equipped with a flame-ionization detector (FID) and Jetanizer™ (Activated Research Company) enabling the determination of $CO_2$.
Synthetic air 5.0 (Siad) was mixed with acetaldehyde (Siad) using two mass flow controllers (Aalborg) to receive the initial concertation of 5 ppm. The synthetic air was divided to a wet and a dry flow and the humidity was adjusted to 50% using two needle valves, measured by a humidity meter. The gas mixture was not allowed to flow through the reactor chamber before the initial concentration of 5 ppm acetaldehyde was reached using two three-way valves (by-pass). After reaching the initial concertation of 5 ppm, the gas flow was passed through the reactor chamber under dark conditions to determine potential adsorption. After reaching a stable acetaldehyde concertation the UV light was turned on.

Table S1. EDX elemental analysis of Nb and Ti content of the non-anodized Ti-Nb alloy meshes.

| | Ti alloy | | |
|---|---|---|---|
| | Ti-1Nb | Ti-5Nb | Ti-10Nb |
| Ti content / % | 99.2 ± 0.1 | 96.4 ± 0.1 | 94.2 ± 0.2 |
| Nb content / % | 0.8 ± 0.1 | 3.6 ± 0.1 | 5.8 ± 0.2 |

Table S2. Atomic concentration of Ti, O, and C species deduced by XPS HR spectra within the TNT layers.

| Ti alloy | Atomic concentration / % | | |
|---|---|---|---|
| | Ti | O | C |
| Ti | 22.3 | 48.5 | 29.1 |
| Ti-1Nb | 23.8 | 52.3 | 23.9 |
| Ti-5Nb | 22.8 | 50.1 | 27.9 |
| Ti-10Nb | 20.6 | 46.7 | 32.8 |

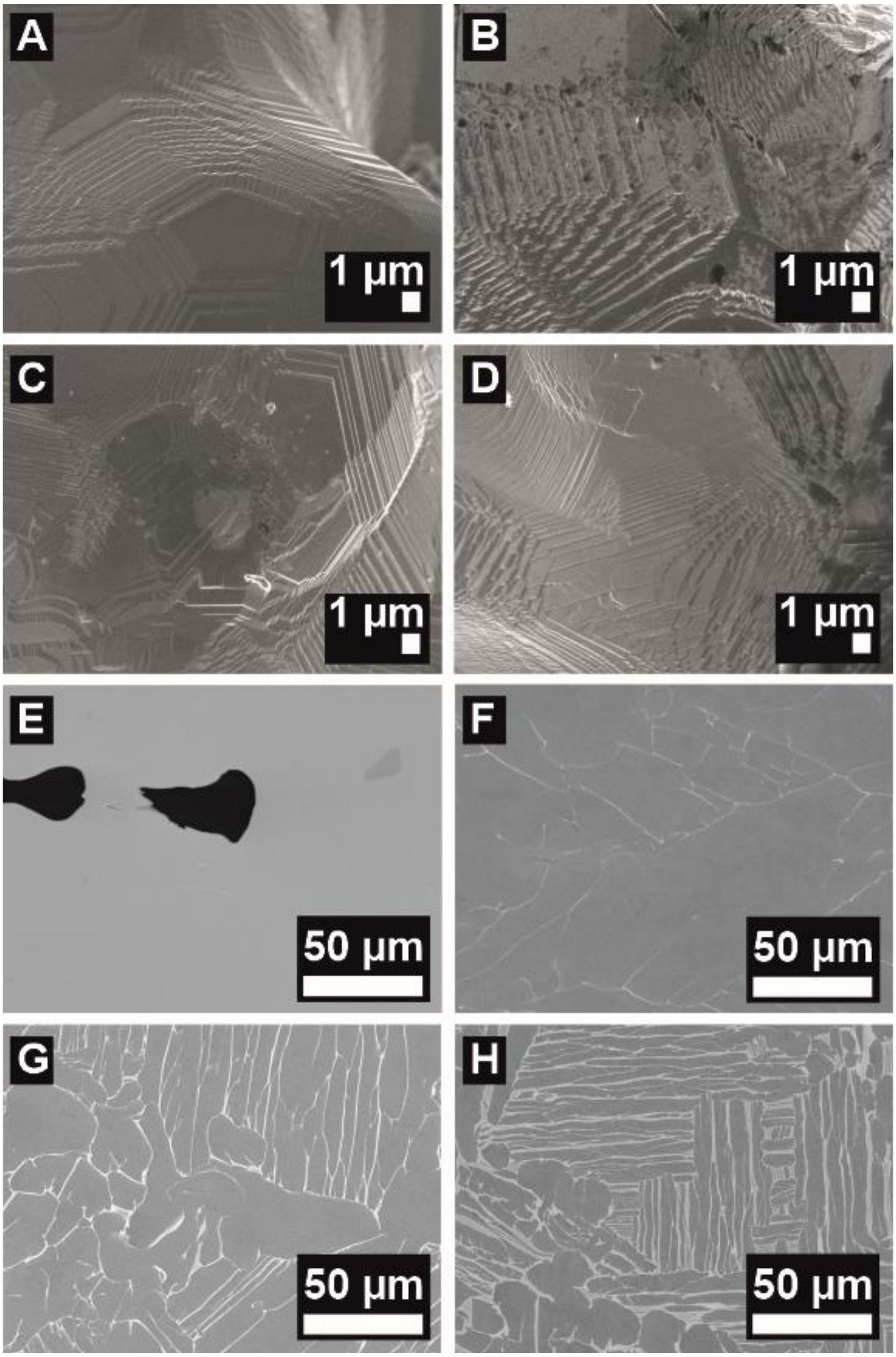


**Figure S1.** SEM images of the non-anodized 3D Ti and TiNb alloys meshes, top – external surfaces A) Ti, B) Ti-1Nb, C) Ti-5Nb, and D) Ti-10Nb, and bottom – metallographic cross-sections E) Ti, F) Ti-1Nb, G) Ti-5Nb, and H) Ti-10Nb.

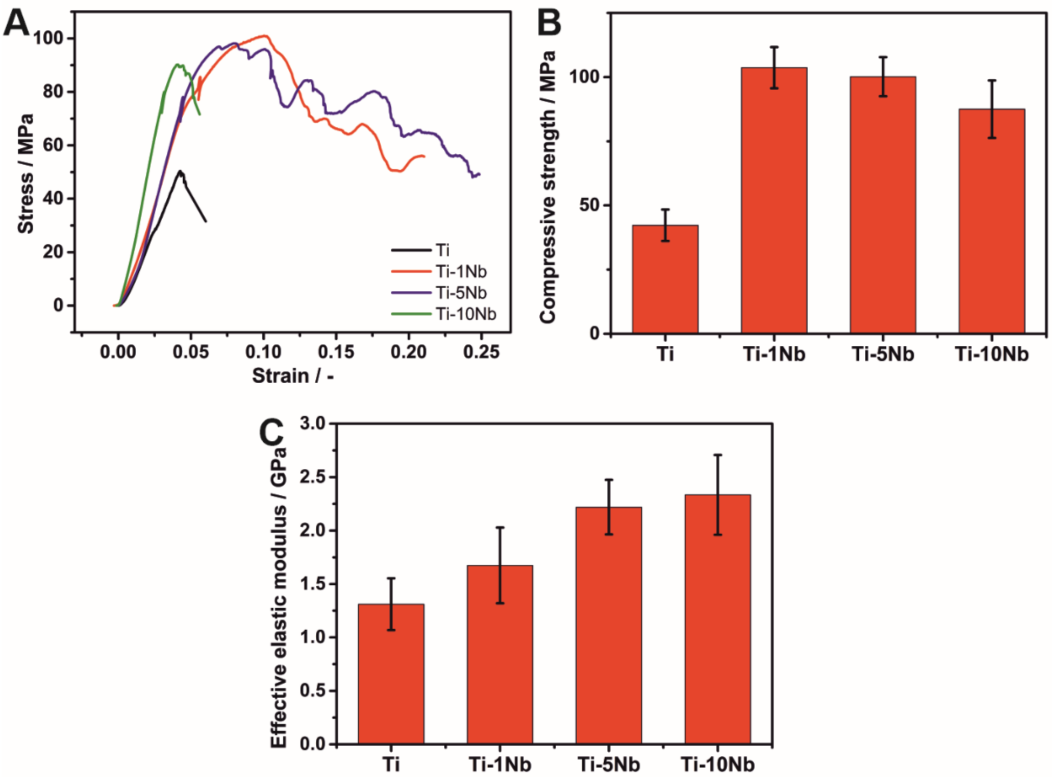


**Figure S2.** Mechanical properties of the 3D Ti and TiNb alloy meshes: A) representative stress-strain curves, B) compressive strength, and C) effective elastic modulus in compression.

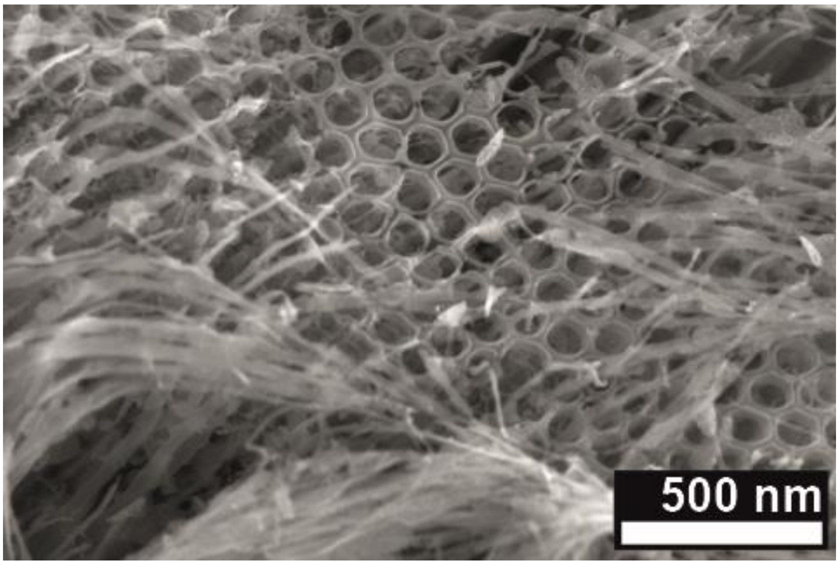


**Figure S3.** SEM top-view image of an anodized 3D Ti-5Nb mesh with nanograss on top of the TNT layer.

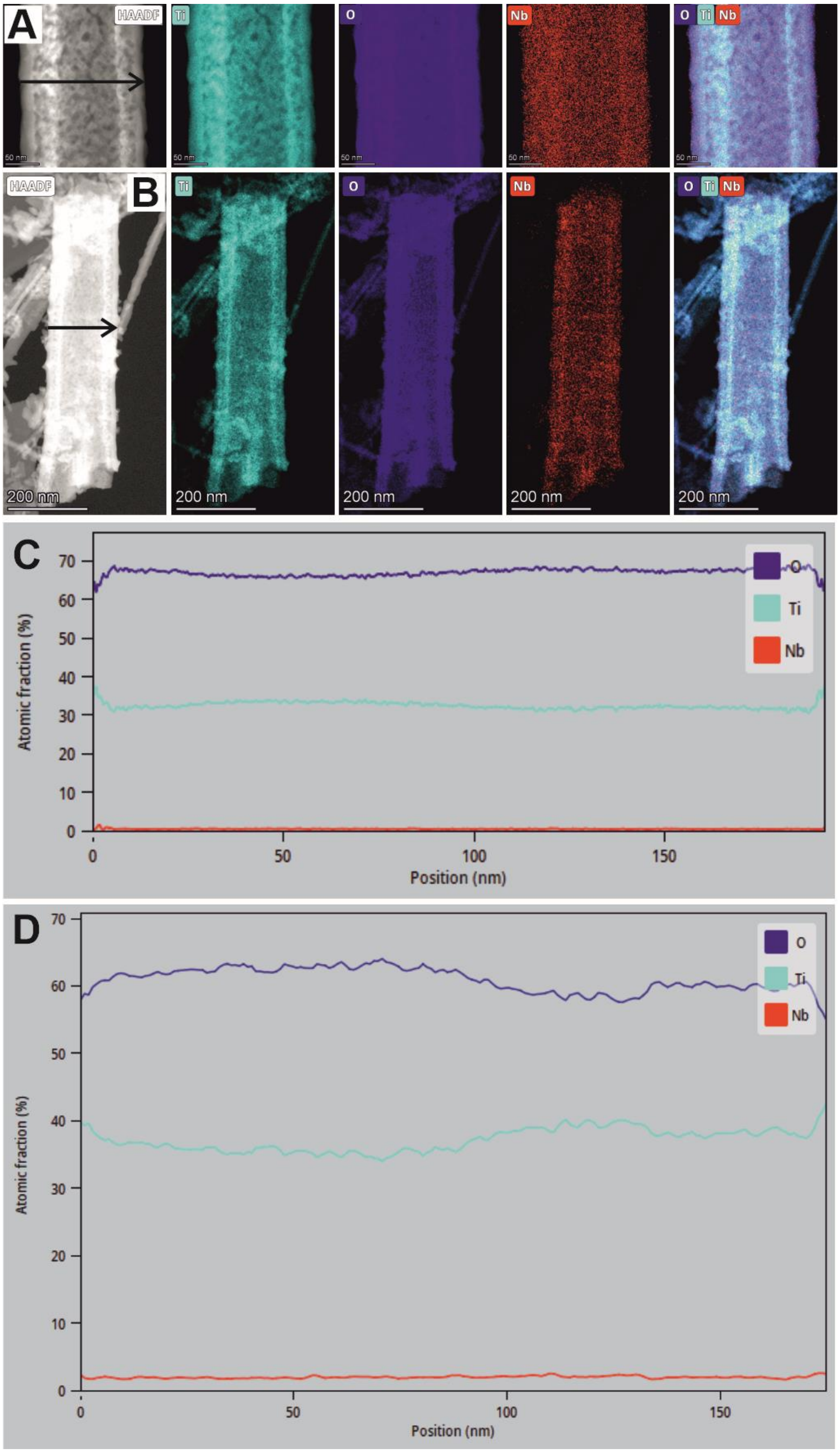


Figure S4. HAADF-STEM images and STEM EDX elemental maps with the distribution of Ti, O and Nb of fragments of $TiO_2$ nanotubes grown on a A) Ti-1Nb mesh, and B) Ti-10Nb mesh. C) and D) show the corresponding EDX maps along the intersection of the nanotube fragments grown on a C) Ti-Nb mesh and D) Ti-10Nb mesh, as indicated by arrows in the HAADF-STEM images.

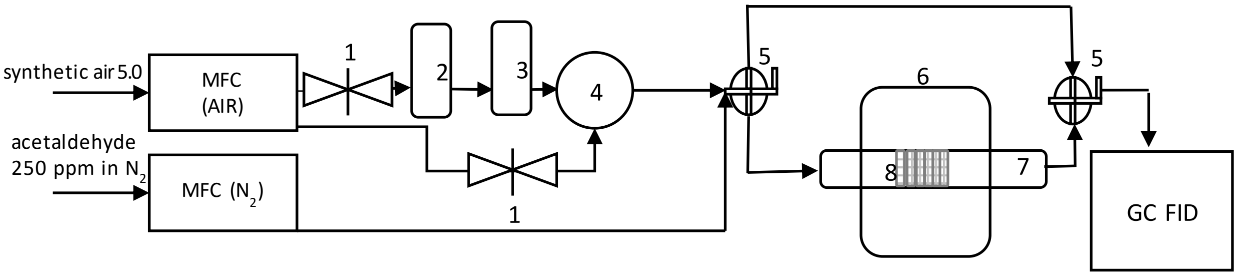


**Figure S5.** Scheme of the used photocatalytic reactor (1 - needle valve, 2 - humidifying flask, 3 - empty flask, 4 - humidity meter, 5 - three valve vent, 6 – UV light source, 7 - quartz tube, 8 - 3D meshes, MFC-mass flow controller, GC FID –gas chromatograph with flame-ionization detector).

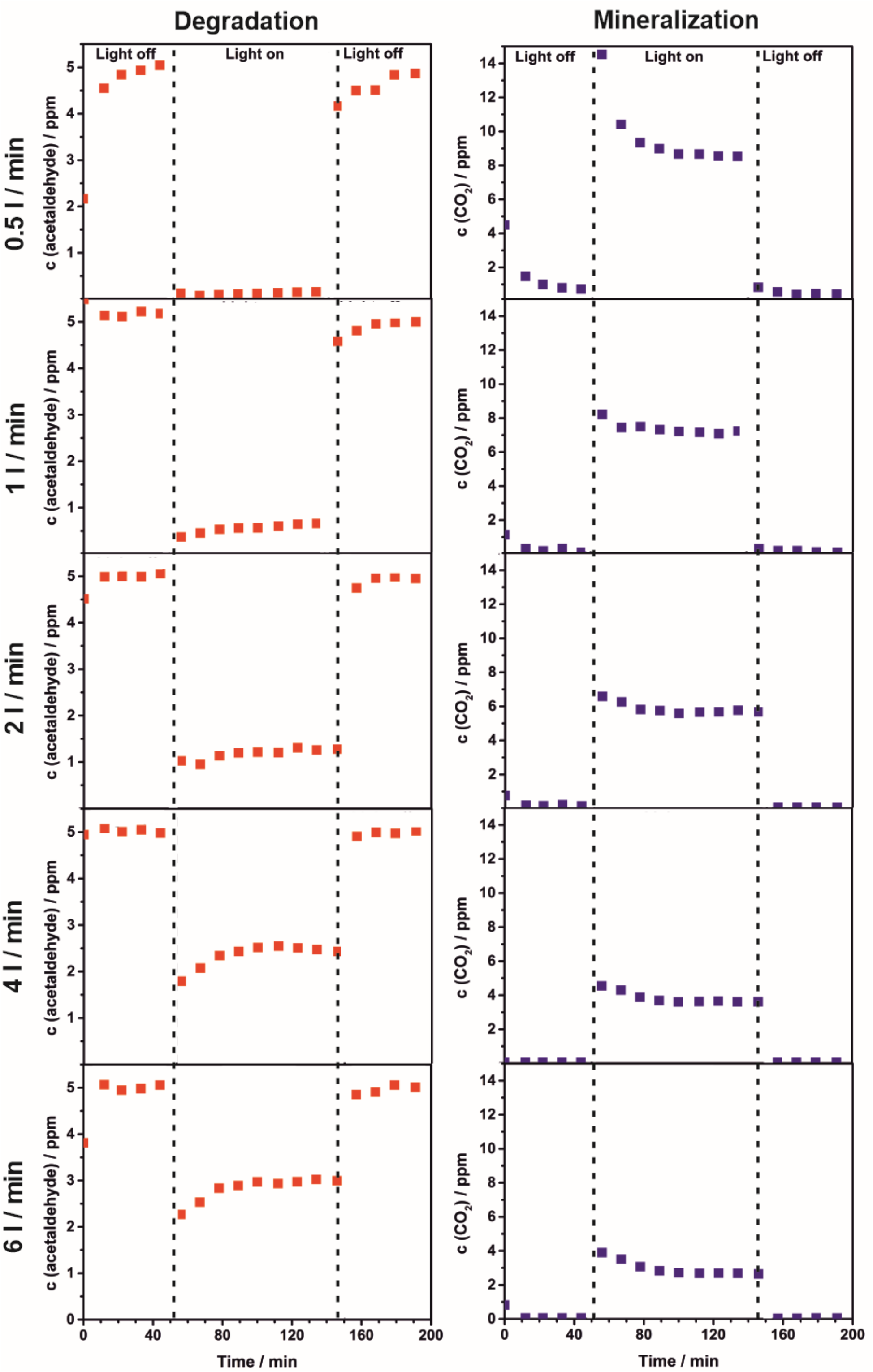


**Figure S6.** Photocatalytic degradation and mineralization of acetaldehyde using 3D Ti meshes as photocatalyst for different flow rates of acetaldehyde in the reactor.

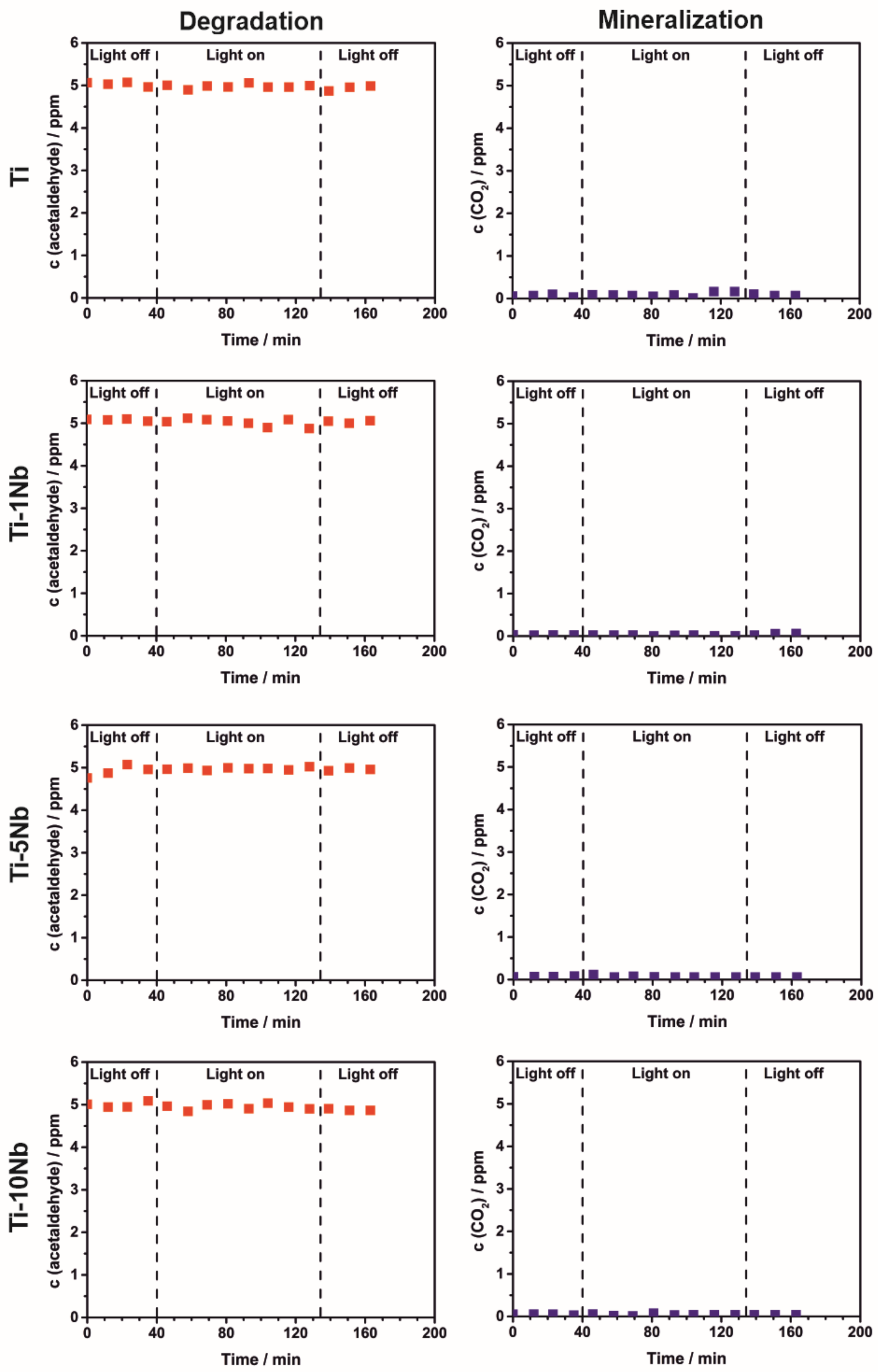


**Figure S7.** Photocatalytic degradation and mineralization of acetaldehyde on annealed 3D Ti and TiNb alloy meshes without TNT layers. Flow rate 4 l / min.

**References**

(1) Montufar, E. B.; Tkachenko, S.; Casas-Luna, M.; Škarvada, P.; Slámečka, K.; Diaz-de-la-Torre, S.; Koutný, D.; Paloušek, D.; Koledova, Z.; Hernández-Tapia, L.; Zikmund, T.; Čelko, L.; Kaiser, J. Benchmarking of Additive Manufacturing Technologies for Commercially-Pure-Titanium Bone-Tissue-Engineering Scaffolds: Processing-Microstructure-Property Relationship. *Addit. Manuf.* **2020**, *36* (July), 101516. https://doi.org/10.1016/j.addma.2020.101516.

(2) Sopha, H.; Kashimbetova, A.; Hromadko, L.; Saldan, I.; Celko, L.; Montufar, E. B.; Macak, J. M. Anodic TiO 2 Nanotubes on 3D-Printed Titanium Meshes for Photocatalytic Applications. *Nano Lett.* **2021**, *21* (20), 8701–8706. https://doi.org/10.1021/acs.nanolett.1c02815.

(3) Smith, M.; Scudiero, L.; Espinal, J.; McEwen, J.-S.; Garcia-Perez, M. Improving the Deconvolution and Interpretation of XPS Spectra from Chars by Ab Initio Calculations. *Carbon N. Y.* **2016**, *110*, 155–171. https://doi.org/10.1016/j.carbon.2016.09.012.
